# Continuing Professional Development – Radiation Therapy

**DOI:** 10.1002/jmrs.70036

**Published:** 2025-11-20

Maximise your continuing professional development (CPD) by reading the following selected article and answering the five questions. Please remember to self‐claim your CPD and retain your supporting evidence. Answers will be available via the QR code and published in JMRS—Volume 73, Issue 4, December 2026.

## Planning for Preservation: Feasibility of Erectile Tissue Sparing During Prostate Stereotactic Radiotherapy

Felicity Hudson, Glen Dinsdale, Tony Young, Anne McMaster, Tania Erven, Olivia Ryan, Sankar Arumugam, Theresa Nguyen, Mark Sidhom, https://doi.org/10.1002/jmrs.70008.

1. What is the estimated number of Australian men diagnosed with prostate cancer by age 85?
  - 1 in 2
  - 1 in 3
  - 1 in 6
  - 1 in 8
2. According to the article, what is a synthetic CT (sCT)?
  - A CT scan using bulk density overrides for tissue, bone and air
  - A fused image combining CT and MRI into one scan
  - A CT‐equivalent image generated from MRI data
  - A CT‐like image derived from PET data
3. Which erectile tissues had planning constraints applied in this article?
  - Neurovascular Bundles; Internal Pudendal Arteries; Penile Bulb
  - Crura; Neurovascular Bundles; Internal Pudendal Arteries
  - Penile Bulb; Crura; Internal Pudendal Arteries
  - Penile Bulb; Corpus Cavernosa; Neurovascular Bundles
4. Why did this study achieve less internal pudendal artery (IPA) dose reduction than Jaccard et al. (2019)?
  - Lower prescribed dose to the PTV and use of endorectal balloons
  - Outdated planning protocol and low priority for the neurovascular bundles
  - Poor patient compliance and limited planning images
  - Higher CTV dose and prioritisation of neurovascular bundles over IPAs
5. What study limitation was identified in the article?
  - Multiple treatment sites increased result variability
  - New planning system limited in‐depth dose analysis
  - Large prostate volumes (> 100 cc) affected dose sparing evaluation
  - Including crura in planning complicated dose assessment

## Answers

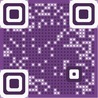

Scan this QR code to find the answers.
